# Anti-chondroitin sulfate proteoglycan 4-specific antibodies modify the effects of vemurafenib on melanoma cells differentially in normoxia and hypoxia

**DOI:** 10.3892/ijo.2015.3010

**Published:** 2015-05-18

**Authors:** DANIELA PUCCIARELLI, NINA LENGGER, MARTINA TAKACOVA, LUCIA CSADEROVA, MARIA BARTOSOVA, HEIMO BREITENEDER, SILVIA PASTOREKOVA, CHRISTINE HAFNER

**Affiliations:** 1 Department of Pathophysiology and Allergy Research, Center for Pathophysiology, Infectiology and Immunology, Medical University of Vienna, Vienna, Austria; 2 Institute of Virology, Department of Molecular Medicine, Slovak Academy of Sciences, Bratislava, Slovakia; 3 Karl Landsteiner Institute for Dermatological Research, St. Poelten, Austria

**Keywords:** melanoma, chondroitin sulfate proteoglycan 4, hypoxia, vemurafenib, hypoxia-inducible factor 1α

## Abstract

Chondroitin sulfate proteoglycan 4 (CSPG4), a highly immunogenic melanoma tumor antigen, is a potential target for antibody-based immunotherapy. The mechanism by which CSPG4 affects melanoma progression is only partly understood, in particular the involvement of other receptor tyrosine kinases and the tumor microenvironment. We have previously reported on a mimotope-based vaccine against CSPG4 in a human melanoma xenograft model that resulted in reduction of tumor growth. Herein we describe the influence of hypoxia on the response to polyclonal anti-CSPG4-antibodies induced by this vaccine in combination with the BRAF inhibitor vemurafenib to enhance therapeutic efficacy by simultaneously targeting multiple signaling pathways. Melanoma cells were treated with polyclonal anti-CSPG4-antibodies and vemurafenib. Proliferation, migration and invasion were evaluated in a real-time setting in the impedance-based x-CELLigence^®^ system. Western blotting and quantitative PCR arrays were used to determine protein and mRNA expression of hypoxia inducible factor 1α (HIF1α), carbonic anhydrase IX (CAIX) and signaling pathway proteins. A melanoma xenograft model was used to detect HIF1α and CAIX expression *in vivo*. Hypoxia enhanced the antiproliferative response to vemurafenib. The migration and invasion capacities of vemurafenib-treated melanoma cells were increased, in spite of vemurafenib-decreased expression of HIF1α and CAIX. Polyclonal anti-CSPG4-antibodies reduced the Transwell migration of vemurafenib-treated, BRAF V600E-mutant and CSPG4-expressing melanoma cells in hypoxia. This was associated with the downregulation of phosphorylated AKT, a kinase contributing to tumor cell migration. Our results highlight CSPG4 as a potential target for modulating treatment resistance to vemurafenib induced by the hypoxic microenvironment.

## Introduction

The incidence of melanoma has increased over the past decades and this tumor entity is responsible for the majority of skin cancer-related deaths (1,2). Melanoma is one of the most challenging types of cancer to be treated due to its high heterogeneity. Surgery remains the main treatment option for early-stage melanomas (3). However, the prognosis of patients diagnosed with metastatic melanoma is still poor, with only a limited number of agents available for treatment (4,5). One of the latest successful drugs for the treatment of advanced melanoma is the BRAF inhibitor vemurafenib (also known as PLX4032) that showed an increase in overall survival and an extension of progression-free survival (6,7). However, incomplete response and acquired drug resistance caused by multiple abnormally regulated signaling pathways present in melanoma frequently led to treatment failures and tumor progression (8–10). Therefore, simultaneously targeting multiple signaling pathways has great potential for enhancing therapeutic efficacy and overcoming resistance to vemurafenib (10,11).

The chondroitin sulfate proteoglycan 4 (CSPG4) is a cell-surface proteoglycan involved in the activation of several signaling pathways playing an important role in tumor cell proliferation, survival, and migration as well as in tumor progression (12). Since it is highly expressed on tumor cells and has restricted distribution in normal tissues, CSPG4 has been used as a target of antibody-based immunotherapy (13,14). The CSPG4-specific monoclonal antibody 225.28 was shown to induce regression of tumor metastases, to inhibit spontaneous metastasis and tumor recurrence in breast cancer mouse models (15), and to inhibit the growth and recurrence of melanoma in a human melanoma xenograft model (16). Recent findings have shown that the combination of vemurafenib and the CSPG4-specific mAb 225.28 was capable of blocking the FAK and PKCα signaling pathways which are important for cell growth, migration, and survival (17). Antibodies induced by a mimotope vaccine (anti-225D9^+^-TT Abs) against the epitope defined by the mAb 225.28 were capable of inhibiting melanoma cell growth *in vitro* and *in vivo* (18,19).

As many other cancers, melanomas include regions of hypoxia caused by an imbalance between oxygen supply and consumption. The response to treatment is affected by this microenvironmental factor (20,21). Tumor hypoxia can negatively influence treatment outcome and patient survival in various cancer types (22,23). Melanomas appear to down-regulate signaling pathways associated with proliferation in order to migrate (24). Hypoxia has been shown to enhance the cell migratory propensity and invasiveness and to contribute to cancer metastasis (25) through the hypoxia inducible factor 1α (HIF1α). HIF1α regulates genes that are regarded pro-tumorigenic (26,27) and increases the expression of a number of genes involved in invasion (28). Carbonic anhydrase IX (CAIX), a direct transcriptional target of HIF1α, plays an important role in maintaining pH homeostasis (29). Earlier studies have shown that a BRAF V600E mutation increased HIF1α expression under hypoxic conditions (30). Hypoxia also induced phenotypic plasticity and therapy resistance in melanoma cells via tyrosine kinase receptors (21).

Herein we report on the response of CSPG4-specific anti-225D9^+^-TT Abs to enhance the anti-proliferative effects of vemurafenib in normoxia. We also describe the role of hypoxia on the response to vemurafenib and anti-225D9^+^-TT Abs and its effect on the anti-proliferative, migratory and invasive potential of melanoma cells.

## Materials and methods

### Cell lines, BRAF inhibitor and polyclonal antibodies

The human CSPG4 expressing (CSPG4^+^) melanoma cell line 518A2 and the human CSPG4 negative (CSPG4^−^) melanoma cell line M14, both harboring the V600E BRAF mutation, were described elsewhere (17,18). Routine tests to exclude mycoplasma and characterize the origin of the cells (short tandem repeat analysis) were performed. Vemurafenib (PLX4032, Selleckchem, Houston, TX, USA) is a potent selective inhibitor of BRAFV600. Polyclonal anti-225D9^+^-TT Abs recognizing CSPG4 were developed and characterized as previously described (18,19). Isotype control anti-TT Abs were used as negative control. Exposure to hypoxia was performed in an anaerobic work station (Ruskin Technologies, Bridgend, UK) in 2% O_2_, 5% CO_2_, 10% H_2_, and 83% N_2_ at 37°C.

### Cell proliferation assays in normoxic and hypoxic conditions

The impedance-based x-CELLingence system (ACEA Bioscience Inc., San Diego, CA, USA) was placed at 37°C in a humidified 5% CO_2_ incubator. 518A2 cells (5×10^3^) were seeded in each well and placed in the x-CELLingence system and proliferation was measured for 24 h. After 24 h the following compounds were added: i) 5 μM vemurafenib, ii) 1 mM DMOG [dimethyloxalylglycine, N-(methoxyoxoacetyl)-glycine methyl ester] (Sigma-Aldrich, St. Louis, MO, USA), and iii) 5 μM vemurafenib plus 1 mM DMOG. The plate was placed back in the x-CELLingence system and measured for 100 h. DMOG was used to induce hypoxia in cells when it was technically not possible to use a hypoxia chamber.

### Cell proliferation with anti-225D9^+^-TT Abs and vemurafenib

In order to determine the optimal doses of vemurafenib on 518A2 and M14 melanoma cell lines, dose-titration experiments were performed. 518A2 (CSPG4^+^) and M14 (CSPG4^−^) cells (2×10^3^) per well were seeded and vemurafenib was added at different concentrations (0.001, 0.01, 0.1, 0.5, 1.0, 10.0 μM). A [^3^H]-Thymidine incorporation assay was performed and percentage of inhibition of proliferation was calculated by comparing the CPM values of treated cells with those of untreated cells, which were set at 100%.

To test the combinatorial treatment of vemurafenib and anti-225D9^+^-TT Abs (2×10^3^) 518A2 (CSPG4^+^) and M14 (CSPG4^−^) cells per well were seeded and incubated with anti-225D9^+^-TT Abs or isotype control anti-TT Abs at a concentration of 200 μg/ml. A [^3^H]-Thymidine incorporation assay was performed as previously described (18). To test the long-term effect of vemurafenib combined with anti-225D9^+^-TT Abs, an 8-day proliferation assay was performed. 518A2 (CSPG4^+^) and M14 (CSPG4^−^) cells (2×10^3^) were seeded. After 24 h, cells were treated with 1 μM vemurafenib, 1 μM vemurafenib plus 200 μg/ml anti-225D9^+^-TT Abs, or 1 μM vemurafenib plus 200 μg/ml isotype control TT Abs. On day 4, 6 and 8 a [^3^H]-Thymidine incorporation assay was performed.

### Transwell migration and Transwell invasion assays

The CIM-Plate 16 (8-μm pore diameter; ACEA Bioscience Inc.) with or without matrigel [1/10 in medium with 10% FCS (BD Biosciences, Franklin Lakes, NJ, USA)], was used for Transwell invasion or migration assays (31). 518A2 cells (4×10^5^) were seeded in quadruplicates and treated with i) serum-free medium (SFM), ii) medium supplemented with 10% fetal calf serum (FSC), iii) vemurafenib (1 μM), and iv) vemurafenib (1 μM) plus anti-225D9^+^-TT Abs (200 μg/ml). The plates were placed in the RTCA DP Analyzer (ACEA Bioscience Inc.) in normoxia or hypoxia and numbers of migrated or invaded cells measured for 48 h. Cell index values are directly proportional to the measured impedances which are automatically and continuously recorded by the RTCA DP instrument.

### Determination of extracellular pH and oxygen consumption in melanoma cells

Since extracellular pH regulation and oxygen consumption are affected by hypoxia, which induces a cell-type specific shift in glycolysis and thereby alters the consumption of oxygen (32), a SDR optical sensor system (1450 MicroBeta TriLux; Perkin-Elmer, Waltham, MA, USA) embedded in the hypoxic box was used for measuring these two parameters. 518A2 (CSPG4^+^) and M14 (CSPG4^−^) cells (0.2×10^6^) were seeded and incubated with i) 1 μM vemurafenib, ii) 1 μM vemurafenib plus 200 μg/ml anti-225D9^+^-TT Abs, and iii) 200 μg/ml anti-225D9^+^-TT Abs. The plates were placed in the hypoxia chamber with 2% of atmospheric oxygen and the extracellular pH and oxygen consumption were measured by using the SDR optical sensor system. The sensor dish reader monitors the pH in real-time in an OxoDish^®^ for oxygen and in a HydroDish^®^ for pH using a non-invasive technique that detects the luminescence lifetime of a sensor spot at the bottom of each well that is dependent on the pH of the surrounding sample. The extracellular pH was measured by the SDR every 25 min for 112 h and the oxygen consumption was measured by the SDR every 5 min for 5 h.

### RNA extraction and quantitative reverse transcription-PCR

Total RNA was extracted with the TRIzol reagent (Sigma-Aldrich) from cells that had been treated with vemurafenib (1 μM) and cultured in hypoxic or normoxic conditions for 24 h. Three micrograms of RNA were reverse-transcribed into cDNA with High-Capacity cDNA Reverse Transcription kit (Applied Biosystems, Foster City, CA, USA) using random heptameric primers. Quantitative RT-PCR analysis of mRNAs of *HIF1α, CA9* and *β-actin* as internal standard were performed on a StepOne™ Real-time PCR System (Applied Biosystems) using Power SYBR^®^ Green PCR Master mix (Applied Biosystems). The following primers were used: *HIF1α* sense: 5′-GCTTGGTGCTGATTTGTGAACC-3′, *HIF1α* antisense: 5′-GCATCCTGTACTGTCCTGTGGTG-3′; *CA9* sense: 5′-CCGAGCGACGCAGCCTTTGA-3′, *CA9* antisense: 5′-GGCTCCAGTCTCGGCTACCT-3′; *β-actin* sense: 5′-TCC TCCCTGGAGAAGAGCTA-3′, *β-actin* antisense: 5′-ACATC TGCTGGAAGGTGGAC-3′. The results were analyzed using the Applied Biosystems 7500 system v1.4.0 software.

### Immunoblot analysis

518A2 melanoma cells were incubated with vemurafenib (1 μM), anti-225D9^+^-TT Abs (200 μg/ml) or vemurafenib (1 μM) plus anti-225D9^+^-TT Abs (200 μg/ml) and placed in normoxia or hypoxia for 24 h. After treatment cells were lysed and equal amounts of proteins were separated by SDS-PAGE under reducing conditions and transferred onto polyvinylidene fluoride (PVDF) membranes (Immobilon Millipore, Billerica, MA, USA). Cells were lysed in lysis buffer [1% Triton X-100, 150 mM NaCl, 50 mM Tris, pH 7.5, 0.5% Nonidet P-40, 50 mM NaF, protease inhibitor cocktail (Roche, Mannheim, Germany) or 10 mM Tris/HCl pH 8.2, 1% NP40, 1 mM EDTA, 150 mM NaCl, protease inhibitor cocktail (Roche)]. Primary antibodies used recognized human CAIX [M75, (33)], HIF1α (BD Transduction Laboratories, San Jose, CA, USA), pFAK(Tyr397), FAK, PKCα (Abcam, Cambridge, UK) and pAKT (Ser473), AKT, pERK 1/2(Thr202/Tyr204), ERK1/2 or β-actin mAbs (Cell Signaling Technology, Danvers, MA, USA). Corresponding peroxidase-conjugated secondary mAbs (Cell Signaling Technology) were used. Blots were developed using the LumiGLO chemiluminescencent substrate (Cell Signaling Technology) and bands visualized using the Filmentwickler CP1000 Processor (AGFA, Mortsel, Belgium).

### Immunohistochemistry in xenografts

The human melanoma C.B.17 SCID/SCID mouse xenotransplantation model was described elsewhere (16). All experiments were approved by the Animal Experimentation Committee of the University of Vienna and the Ministry of Education, Science and Culture, Vienna, Austria. Untreated 518A2 melanoma tumors were fixed in 4% paraformaldehyde and embedded in paraffin. For CAIX detection, sections were stained in the Dako Autostainer using the DakoCytomation EnVision^®^+ System-HRP (Dako, Glostrup, Denmark) according to the manufacturer’s instructions. Antibodies used were the anti-CAIX antibody M75 and a secondary anti-mouse IgG antibody (Dako). Staining was visualized with DAB solution (Dako). For HIF1α immunostaining, antigen retrieval was carried out with citrate buffer, pH 6.0, for 5 min at 125°C using a Pascal pressure chamber (Dako). Deparaffinized sections were stained with DakoCytomation Catalysed Signal Amplification System according to the manufacturer’s instructions. Sections were incubated with a primary antibody specific for HIF1α (1:250; BD Transduction Laboratories). Staining was visualized with DAB solution. The stained sections were examined with an Olympus BX53 microscope and photographed with an Olympus DP73 camera (Olympus Electronics, Tokyo, Japan).

## Results

### Vemurafenib inhibits cell growth of BRAF V600E mutant 518A2 melanoma cells in normoxic and hypoxic conditions

**Figure 1:**
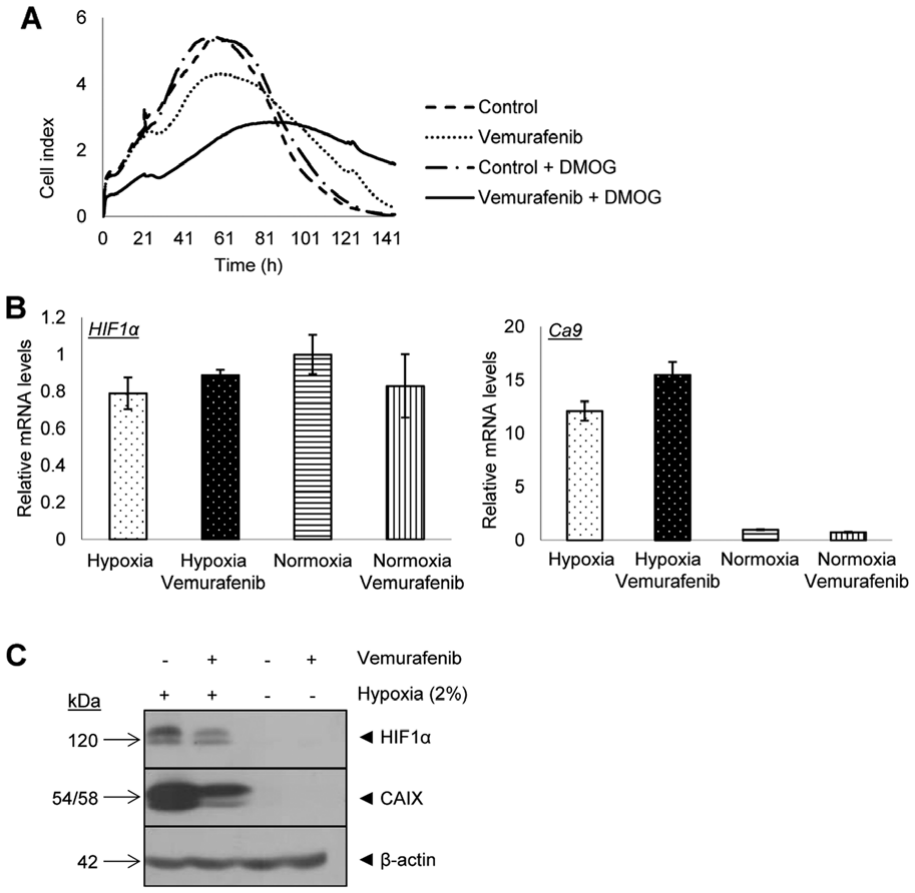
Hypoxia influences the response of vemurafenib in 518A2 melanoma cells and effects mRNA levels and protein expression of HIF1α and CAIX. (A) Time lapse measurements of the inhibitory effect of vemurafenib in normoxic and hypoxic conditions by the x-CELLigence system. (B) Real-time PCR analysis of *HIF1α* and *CA9* mRNA levels, normalized to the internal control (*β-actin*). Error bars represent ± SD from three different experiments. (C) Expression of HIF1α and CAIX proteins in vemurafenib-treated 518A2 cells was evaluated by immunoblot analyses.

Treatment of melanoma cells with increasing concentrations of vemurafenib resulted in a dose-dependent inhibition of proliferation of the BRAF V600E mutant melanoma cell lines 518A2 and M14. The growth of the BRAF V600E mutant 518A2 melanoma cells and M14 melanoma cells treated with 1 μM vemurafenib was inhibited by 40±3% and 60±5%, respectively (data not shown). In order to visualize the time-course of the inhibitory effect of vemurafenib, a real-time characterization was performed using the x-CELLingence system. 518A2 cells were treated with 5 μM vemurafenib and cell proliferation was measured for up to 130 h in normoxic and hypoxic conditions. 518A2 cells were not susceptible to hypoxic conditions and the proliferation rate did not change (maximum cell index of 5.3). After addition of vemurafenib, hypoxic 518A2 cells (maximum cell index of 2.8) reduced cell growth by an additional 38% compared to normoxic, vemurafenib-treated 518A2 cells (maximum cell index of 4.3) (Fig. 1A). The inhibitory peak after vemurafenib in normoxic conditions was reached after 60 h, whereas hypoxia shifted the peak to 90 h (Fig. 1A).

### Effect of hypoxia on HIF1α and CAIX protein expression and mRNA levels in vemurafenib-treated 518A2 melanoma cells

To investigate how hypoxia influences the response of 518A2 cells to vemurafenib, we focused on important markers of hypoxia, HIF1α and CAIX. Messenger RNA levels of *HIF1α* did not show significant changes. The relative mRNA level of HIF1α in 518A2 cells in hypoxia was 0.8±0.08. Treatment with vemurafenib increased the mRNA level of *HIF1α* to 0.9±0.02. In normoxic conditions the relative mRNA levels of *HIF1α* were 1.0±0.1 and 0.8±0.17 for untreated and treated 518A2 cells, respectively (Fig. 1B). The relative *CA9* mRNA levels were 1.0±0.5 in normoxic conditions with and without vemurafenib treatment. In hypoxic conditions *CA9* mRNA levels increased to 12.0±0.9 for untreated cells and to 16.0±1.1 for vemurafenib-treated cells (Fig. 1B). All mRNA levels have been normalized to the housekeeping gene coding for *β-actin*. Both proteins were expressed in hypoxic 518A2 cells. After treatment with vemurafenib in hypoxic conditions HIF1α and CAIX protein expression was downregulated, whereas in normoxic conditions HIF1α and CAIX were not expressed in either treated or untreated 518A2 cells (Fig. 1C).

### Expression of hypoxia markers in melanoma xenografts

**Figure 2:**
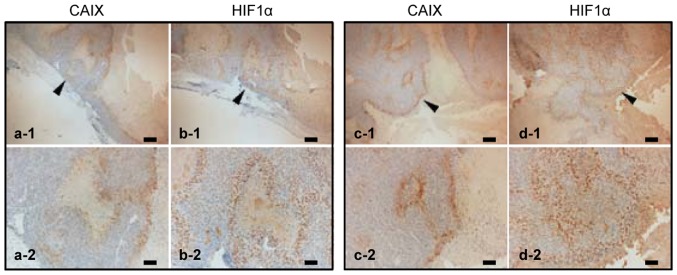
Immunohistochemistry of two representative 518A2 xenograft tumor samples stained with HIF1α and CAIX antibodies from corresponding tumor regions. Overview images of two representative melanoma tumors are shown in the top row (a-1, b-1 and c-1, d-1); staining for CAIX (a-1, a-2, c-1 and c-2) and HIF1α (b-1, b-2, d-1 and d-2) shows regions with high expression. Scale bars, 200 μm (a-1, b-1, c-1 and d-1), and 100 μm (a-2, b-2, c-2 and d-2). Arrows point to the area of magnification.

The expression of HIF1α and CAIX in a 518A2 melanoma xenograft model was evaluated by immunohistochemistry. As shown in Fig. 2, immunohistochemical staining of formalin-fixed, paraffin-embedded tumor tissues demonstrated the presence of HIF1α and CAIX, which showed typical, hypoxia-related expression patterns. These *in vivo* results reflect the expression level of both proteins observed *in vitro*.

### Anti-225D9^+^-TT Abs enhance the effects of vemurafenib in CSPG4 expressing 518A2 melanoma cells

**Figure 3:**
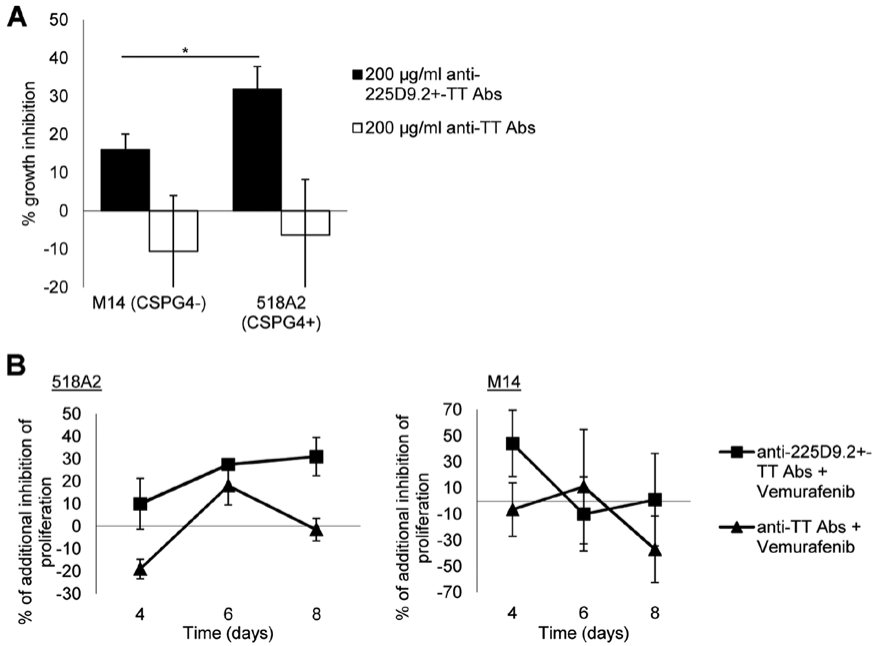
CSPG4-specific anti-225D9^+^-TT Abs enhance the antiproliferative effects of vemurafenib in 518A2 melanoma cells. (A) Growth inhibition of 518A2 melanoma cells [BRAF(V600E)/CSPG4^+^] by anti-225D9^+^-TT Abs compared to M14 melanoma cells [BRAF(V600E)/CSPG4^−^] (^*^P<0.05). (B) Anti-225D9^+^-TT Abs contribute to an additional growth inhibition in vemurafenib-treated 518A2 melanoma cells [BRAF(V600E)/CSPG4^+^] which was not the case in M14 melanoma cells [BRAF(V600E)/CSPG4^−^]. Anti-TT Abs were used as isotype control. Error bars represent ± SD from three different experiments.

Targeting multiple signaling pathways was more effective in suppressing the growth of BRAF V600E mutant melanoma cells (11). Treatment with CSPG4 specific anti-225D9^+^-TT Abs showed that the growth of 518A2 cells [BRAF(V600E)/CSPG4^+^] was inhibited by 32±5.9% compared to M14 cells [BRAF(V600E)/ CSPG4^−^] (Fig. 3A). When combining vemurafenib with anti-225D9^+^-TT Abs, this led to an additional growth inhibition of the 518A2 [BRAF(V600E)/CSPG4^+^] by 30±6.0% on day 8 compared to the CSPG4 negative cell line M14 (Fig. 3B). These results showed that targeting the CSPG4 protein alone and in combination with vemurafenib influenced the proliferation rate of 518A2 melanoma cells.

### Influence of vemurafenib and anti-225D9^+^-TT Abs on the Transwell migration and Transwell invasion capacity of 518A2 melanoma cells

**Figure 4:**
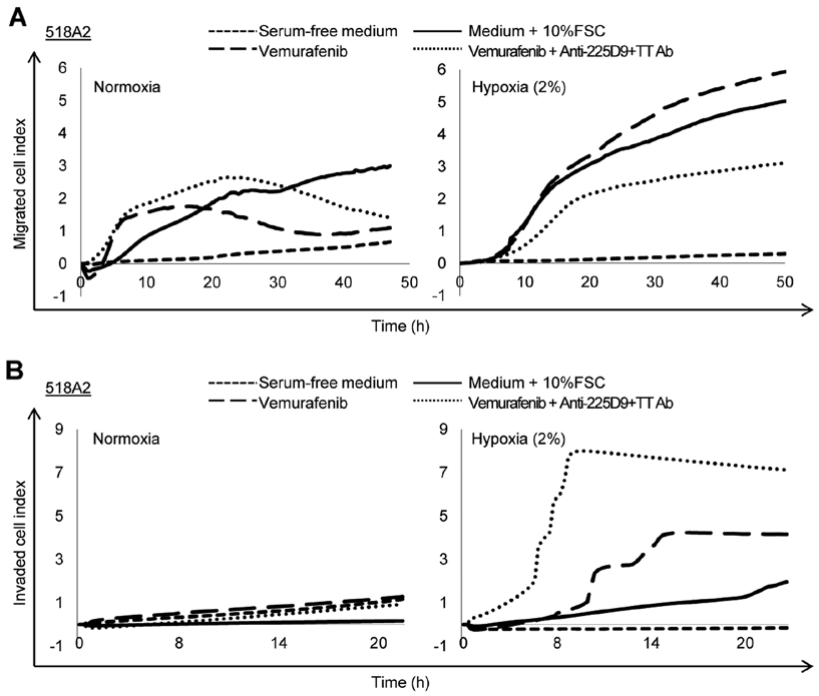
CSPG4-specific anti-225D9^+^-TT Abs decrease Transwell migration but increase Transwell invasion in hypoxic, vemurafenib-treated melanoma cells. (A) Migration capacity of anti-225D9^+^-TT Abs- and vemurafenib-treated 518A2 melanoma cells in normoxic and hypoxic conditions. (B) Invasion capacity of anti-225D9^+^-TT Abs- and vemurafenib-treated 518A2 melanoma cells in normoxic and hypoxic conditions.

In order to measure the tumorigenicity of the 518A2 melanoma cells in hypoxic and normoxic conditions, a Transwell migration assay using the RTCA DP Analyzer was performed (Fig. 4A). In normoxic conditions, untreated 518A2 cells migrated relatively slowly but continuously and reached a cell index of 3.3 after 50 h. In the presence of vemurafenib, the cells initially migrated and reached a cell index of 1.8 after 15 h, and then their migration index decreased to 0.9 after 38 h. The combination of vemurafenib and anti-225D9^+^-TT Abs had a similar effect on the course of migration, since the cell index initially increased to 2.6 after 22 h and then decreased to 1.6 after 46 h. As expected, in hypoxic conditions the cell index was markedly increased in untreated 518A2 melanoma cells, where it reached a value of 5.0 after 52 h. The migration capacity of vemurafenib treated melanoma cells was even slightly increased and reached a cell index of 6.0 when compared to untreated cells. In contrast, vemurafenib together with anti-225D9^+^-TT Abs decreased the migration capacity of 518A2 cells, reaching a cell index of 3.0 after 50 h.

To test the invasive potential of 518A2 melanoma cells, a Transwell matrigel invasion assay was performed. In normoxic conditions, untreated 518A2 cells did not show any invasion capacity, reaching a cell index of 0.1 after 22 h (Fig. 4B). Vemurafenib or vemurafenib together with anti-225D9^+^-TT Abs increased the cell indices slightly to 0.85 and 1.4, respectively. However, in hypoxic conditions the invasion capacity of untreated 518A2 melanoma cells increased and the cell index reached 1.4 after 22 h. Surprisingly, when the cells were exposed to vemurafenib or vemurafenib and anti-225D9^+^-TT Abs, the effect was intensified with cell indices reaching 4.0 and 7.2 after 22 h, respectively.

### Anti-225D9^+^-TT Abs influence the metabolism in hypoxic, vemurafenib-treated melanoma cells

**Figure 5:**
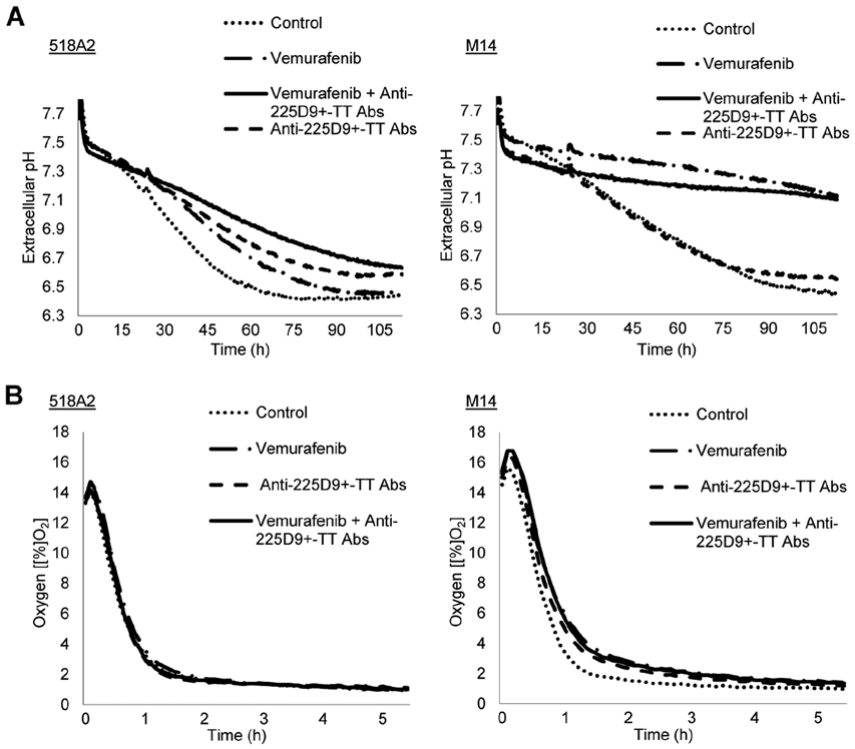
Anti-225D9^+^-TT Abs influence the metabolism in hypoxic, vemurafenib-treated melanoma cells. (A) Effect of vemurafenib and anti-225D9^+^-TT Abs on the extracellular pH regulation in hypoxic 518A2 melanoma cells [BRAF(V600E)/CSPG4^+^] compared to M14 cells [BRAF(V600E)/CSPG4^−^]. (B) Effect of vemurafenib and anti-225D9^+^-TT Abs on the oxygen consumption in hypoxic 518A2 melanoma cells [BRAF(V600E)/CSPG4^+^] compared to M14 cells [BRAF(V600E)/CSPG4^−^].

Untreated 518A2 cells showed a decrease in the extracellular pH. After adding vemurafenib, anti-225D9^+^-TT Abs or a combination of both, the extracellular pH decreased more slowly compared to untreated 518A2 cells (Fig. 5A and Table I). No change was observed in oxygen consumption between treated and untreated 518A2 cells (Fig. 5B). In contrast, no anti-225D9^+^-TT Abs-induced shift in extracellular pH was observed in the CSPG4 negative M14 melanoma cell line (Fig. 5A and Table I). A minor change for oxygen consumption from 2 to 4% was observed in this cell line in all treatment modalities (Fig. 5B).

### Vemurafenib and anti-225D9^+^-TT Abs differently affect multiple signaling pathways in normoxic and hypoxic conditions

**Figure 6:**
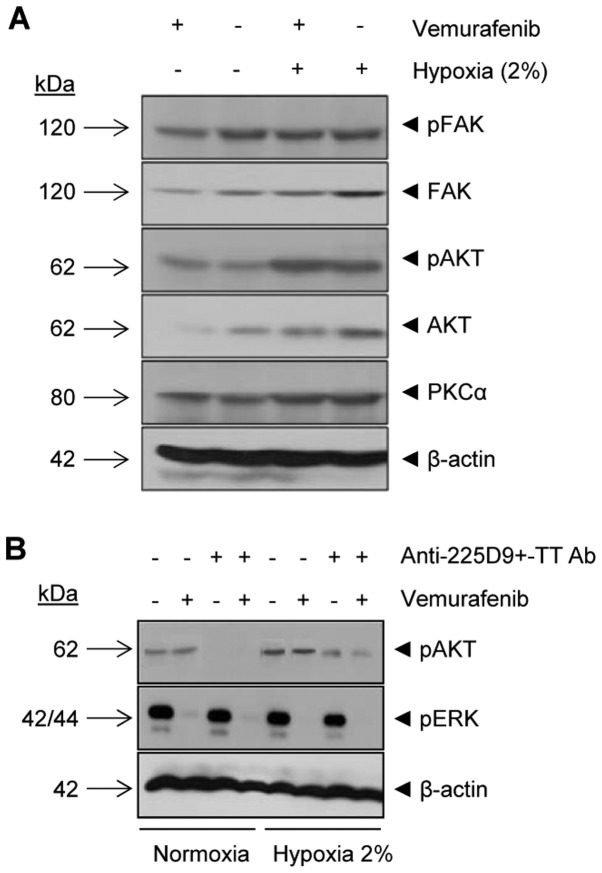
Hypoxia affects multiple signaling pathway proteins in 518A2 melanoma cells treated with vemurafenib and anti-225D9^+^-TT Abs. (A) Immunoblot analyses of proteins involved in PI3K, JAK-STAT and MAPK pathways under normoxic and hypoxic conditions after treatment with vemurafenib. (B) Western blotting of pAKT (Ser473) and pERK1/2 (Thr202/ Tyr204) in 518A2 cells treated with vemurafenib and anti-225D9^+^-TT Abs in normoxic and hypoxic conditions.

To understand the molecular heterogeneity in melanoma we compared the effects on signaling pathways important to cell growth, migration, invasion and survival in 518A2 melanoma cells after treatment with vemurafenib in normoxic and hypoxic conditions. In normoxic conditions, vemurafenib treatment of the melanoma cell line 518A2 resulted in a decreased protein expression level of pERK1/2 (Thr202/Tyr204) (Fig. 6B) and decreased expression levels of pFAK (Tyr397), FAK and AKT compared to untreated 518A2 cells (Fig. 6A). However, in hypoxic conditions untreated melanoma cells showed an increased protein expression of FAK and AKT compared to untreated cells in normoxia. The expression levels of PKCα and pAKT (Ser473) increased in vemurafenib-treated and untreated hypoxic 518A2 cells compared to normoxic cells (Fig. 6A).

To understand the molecular mechanisms underlying the modification of vemurafenib-mediated effects when combined with anti-225D9^+^-TT Abs, AKT and ERK signaling pathways were analyzed. The phosphorylation level of AKT (Ser473) increased in hypoxic conditions. However, pAKT (Ser473) slightly decreased after the treatment anti-225D9^+^-TT Abs and was decreased even more when vemurafenib was combined with anti-225D9^+^-TT Abs. No change was observed in the phosphorylation level of ERK1/2 (Thr202/Tyr204) (Fig. 6B).

## Discussion

Melanoma is one of the most aggressive skin cancers. The poor efficiency of available therapies demands to find new therapeutic strategies to improve patient survival rates and to overcome resistance to currently used drugs (8,34). Oncogenic mutations within the MAPK pathway are frequent in melanoma and targeting of MAPK signaling has yielded significant responses in a large number of patients that last for several months before relapsing (35).

One of the factors which contributes to melanoma progression is hypoxia through HIF-mediated molecular responses. HIF1 promotes the upregulation of genes which control a series of metabolic changes in tumor cells as well as increasing their invasive properties (36,37). Thereby hypoxia significantly affects tumor phenotype and in many tumor types is associated with therapy resistance (38,39). In melanoma, hypoxia is a microenvironmental stimulus that triggers a switch from a proliferative to an invasive cell phenotype that is less sensitive to therapies (21,26). In our studies, hypoxia did not influence the proliferative capacity of 518A2 melanoma cells, whereas hypoxia enhanced the antiproliferative capacity of vemurafenib in a time-dependent manner (Fig. 1A). One of the mediators of hypoxic responses in many cell types is the HIF1α protein (36,37). Vemurafenib markedly reduced the protein expression of this hypoxic marker in 518A2 melanoma cells and this downregulation also contributed to lower expression levels of CAIX (Fig. 1C). This was not reflected on the mRNA level of HIF1α, suggesting that the transcription of HIF1α is not influenced by vemurafenib (Fig. 1B). Widmer *et al* (26) showed that knock-down of *HIF1α* under hypoxic conditions decreased the invasion capacity of melanoma cells. We demonstrated that reduced HIF1α expression was responsible for enhanced Transwell migration and invasion despite unchanged *HIF1α* mRNA levels (Figs. 1B, and 4). CAIX is a hypoxia-inducible, tumor-associated member of the human α-CA family (40). CAIX shows only limited expression in normal tissues but its expression is highly elevated in various cancers such as colorectal and lung carcinomas (41,42) and has not been shown in melanomas (43). To the best of our knowledge, we demonstrate for the first time CAIX expression and its downregulation by vemurafenib in 518A2 melanoma cells (Fig. 1C). We also observed CAIX staining in 518A2 xenografts in many nested tumor regions, preferable near necrotic areas co-localized with HIF1α (Fig. 2).

Clinical evidence has already shown that melanoma regression is rarely complete after 6–9 months of therapy with vemurafenib (7). This is partly due to acquired resistance that leads to melanoma progression and a boost in aggressiveness of the disease (34) but also to the tumor microenvironment and the fact that melanomas are composed of heterogeneous zones containing proliferative and quiescent cells (44). A combined approach with vemurafenib and inhibitors targeting other signaling pathways determined to be abnormal in melanoma cells would be preferential. Therefore, we combined vemurafenib with antibodies directed against CSPG4 and evaluated their therapeutic potential in normoxic and hypoxic conditions. The CSPG4-specific mAb 225.28S was shown to inhibit the growth and recurrence of melanoma in mice grafted with CSPG4-expressing human melanoma cell lines (16) and the antibodies induced by a mimotope vaccine (anti-225D9^+^-TT Abs) directed against CSPG4 were capable of inhibiting melanoma cell growth in a SCID mouse model (18,19). In line with these data we found that anti-225D9^+^-TT Abs were effective in normoxic conditions against proliferative cells and reduced the growth of human melanoma 518A2 cells by 30% (Fig. 3A). Here we show that the combination of vemurafenib and anti-225D9^+^-TT Abs could reduce the growth of 518A2 cells by additional 30% (Fig. 3B). Comparable results were achieved with the CSPG4-specific mAb 225.28 (17).

This finding provided the rationale for the strategy described in this report that combined vemurafenib with antibodies directed against CSPG4 as a means of enhancing the magnitude of the BRAF inhibitor responses that could also influence migration and invasion. There are varying results regarding the effect of MAPK pathway inhibition on invasion and migration in the literature (44,45). We tracked 518A2 cells in the presence of vemurafenib and anti-225D9^+^-TT Abs for long periods of time within a hypoxic microenvironment and obtained a longitudinal track for invasion and migration. We showed that Transwell migration of 518A2 cells toward a chemoattractant was markedly increased in hypoxic conditions and even more after the treatment with vemurafenib. This effect was partly blocked by anti-225D9^+^-TT Abs (Fig. 4A) reflecting the role of CSPG4 in cell migration (12). However, the addition of anti-225D9^+^-TT Abs markedly enhanced the invasive capacity of vemurafenib-treated, hypoxic 518A2 cells (Fig. 4B).

Our data imply that hypoxia is important in determining the effect of targeting CSPG4 on cell migration and invasion. Since hypoxia is associated with changes in the extracellular pH and related to the acidification of the tumor microenvironment that promotes cancer cell invasion (46), we were able to show that vemurafenib increased the extracellular pH in 518A2 cells, an effect that was diminished by the addition of anti-225D9^+^-TT Abs (Fig. 5A and Table I). This was not reflected in the oxygenation levels (Fig. 5B) underlining the role of CSPG4 irrespective of oxygenation in tumor microenvironment and showing its potential as a therapeutic target. In a next step we focused on the capacity of vemurafenib and anti-225D9^+^-TT Ab to block multiple signaling pathways in hypoxic and normoxic conditions. Kumar *et al* (30) showed that the BRAF inhibitor sorafenib inhibited ERK phosphorylation and suggested that some of the effect of this compound was mediated through HIF1α inhibition. Hypoxia *per se* influenced multiple signaling pathways such as pAKT (Ser473), AKT and PKCα (Fig. 6A). In this study we found that the expression of pAKT (Ser473) decreased after treatment with vemurafenib and anti-225D9^+^-TT Abs in hypoxic conditions (Fig. 6B) confirming that hypoxia affected the response to vemurafenib and anti-225D9^+^-TT Abs in 518A2 melanoma cells.

In this study we showed that targeting CSPG4 in melanoma cells enhanced antiproliferative effects of vemurafenib in normoxic conditions and reduced the migratory capacity in hypoxic conditions. Hypoxia strongly influenced the response to vemurafenib treatment in melanoma cells which switched to a more invasive and aggressive phenotype. Therefore, therapeutic efforts will have to consider that the microenvironment of melanoma cells has an impact on tumor progression.

## Figures and Tables

**Table I:** Extracellular pH measured 75 h after the administration of vemurafenib and anti-225D9^+^-TT Abs to hypoxic 518A2 melanoma cells [BRAF(V600E)/CSPG4^+^] and hypoxic M14 melanoma cells [BRAF(V600E)/CSPG4^−^].

Cells	Control	Vemurafenib	Anti-225D9^+^-TT Abs	Vemurafenib and Anti-225D9^+^-TT Abs
518A2 [BRAF(V600E)/CSPG4^+^]	6.4	6.6	6.7	6.8
M14 [BRAF(V600E)/CSPG4^−^]	6.6	7.3	6.6	7.2

## Abbreviations

CSPG4: chondroitin sulfate proteoglycan 4
HIF1α: hypoxia-inducible factor 1α
CAIX: carbonic anhydrase IX
DMOG: dimethyloxalylglycine, N-(methoxyoxoacetyl)-glycine methyl ester
PVDF: polyvinylidene fluoride
